# Surgery and Anesthesia Management for Intraoral Synechia: A Case Report

**Published:** 2018-03

**Authors:** Mohammad Gharavifard, Iman Kashani, Marjan Joudi, Majid Sharifian, Seyed Javad Sayedi, Behrouz Mohammadipanah, Farideh Jamali-Behnam

**Affiliations:** 1 *Department of Anesthesiology, * *Faculty of Medicine,* * Mashhad University of Medical Sciences, Mashhad, Iran.*; 2 *Cardiac Anesthesia Research Center, Faculty of Medicine, Mashhad University of Medical Sciences, Mashhad, Iran.*; 3 *Surgical Oncology Research Center, Mashhad University of Medical Sciences, Mashhad, Iran.*; 4 *Neonatal Research Center, Mashhad University of Medical Sciences, Mashhad, Iran.*; 5 *Department of General Surgery, Faculty of Medicine, Mashhad University of Medical* * Sciences* *.*

**Keywords:** Anesthesia, Congenital abnormality, Surgery

## Abstract

**Introduction:**

Intraoral synechia is a rare congenital condition, generally associated with other maxillo-facial malformations. We present a neonate with congenital intraoral bilateral synechia without any other facial anomalies.

**Case Report:**

In this paper, we present a 19-day-old male neonate with congenital intraoral bilateral synechia without any other facial anomalies. We review the literature to discuss the surgical and anesthesia management of this rare congenital disease.

**Conclusion:**

The disease manifested with a wide spectrum of symptoms. Most cases need surgery and airway management. In patients with a low risk of bleeding or a compromised airway, it is possible to manage them with face mask-inhalation anesthesia and maintain spontaneous breathing.

## Introduction

Isolated congenital intraoral synechia is a rare malformation (1), with an unknown incidence and etiology (2). It can present as partial or complete fusion of the intraoral soft tissue and may be associated with other maxillo-facial malformations, such as facial hemiatrophy or temporomandibular ankylosis and cleft palate, cleft lip, microglossia, micrognathia, or limb anomalies (3–5). We present a neonate with congenital intraoral bilateral synechia without any other facial anomalies.

## Case Report

A 19-day-old male neonate was brought to our children’s hospital with a complaint of poor feeding. He was born uneventfully by lower segment Cesarean section at a gestational age of 36 weeks. The parents were not relatives. There was no history of congenital anomalies or any genetic diseases. The mother was not smoker, and she did not consume any drugs or alcohol before or during pregnancy.

**Fig 1:**
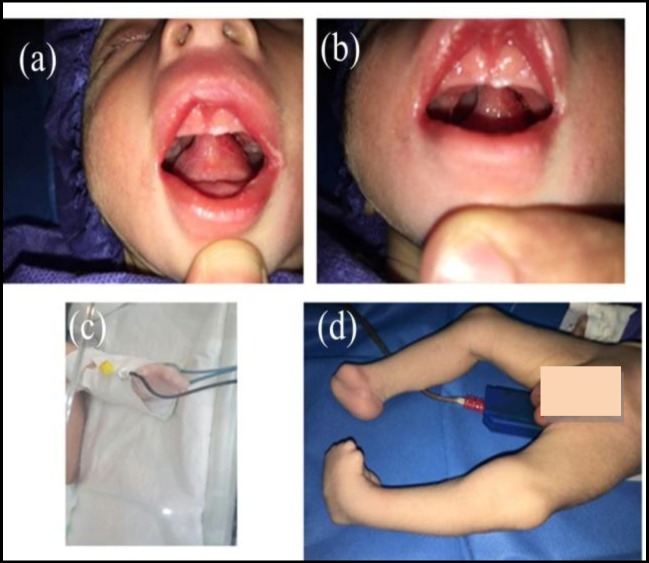
A neonate with congenital intraoral bilateral synechia without any other facial anomalies. a, b) Limited mouth opening. c,d) Undeveloped fingerless hands and rocker-bottom foot

On examination, the patient weighed 2,700 g, and his mouth opening was limited (5 mm) (Fig 1a & b). A thick band at the tip of the tongue adhered to the hard palate in the mid line (Fig. 1). This band was a barrier for the passing of the laryngoscope, tracheal tube or any other supraglottic devices. The patient had deformities in the upper and lower extremities including undeveloped fingerless hands and a rocker-bottom foot (Fig 1c & d). Other systems were normal.

Further investigation confirmed normal karyotype (46, XY), normal echocardiogram parameters and normal laboratory data. Plain chest and skull radiographies showed no abnormalities.

The planned surgery was a simple mucosal band release with electrocautery and short duration. As there was very low risk for bleeding or compromising airway, we planned for inhalation anesthesia with sevoflurane while maintaining spontaneous breathing. Meanwhile, emergency tracheostomy was prepared if needed. Anesthesia was induced with sevoflurane 5% and fentanyl 1 μg/kg, without any muscle relaxants, and was maintained by sevoflurane 2% with oxygen and face mask ventilation through a Mapelson D system. Finally, the band was released from the hard palate with Bovie cautery, the tongue was returned to its normal position and the patient was sent to the pediatric intensive care unit (PICU) for several hours.

## Discussion

Adhesion between intraoral soft tissue structures is defined as synechia (1). The most common form of this anomaly is a connection between the upper and lower jaw, which is called syngnathism. In other cases, the tongue can be connected to the palate, maxilla and floor of the mouth or even the oropharynx.

Fridenberg reported the first case of syndromic congenital synechia in 1908, and to the best of our knowledge, only 25 cases have been reported until now (3). Most reported cases have undergone surgery in the first months after birth due to feeding or respiratory difficulties and failure to thrive; however, there are cases who have undergone surgery in the second or third decades of life (6). Our case was referred because of breast feeding difficulties, as the infant gained only 150 g in the first 3 weeks of life.

Although the exact etiology of synechia has been debated, two prominent theories have been proposed. First is the close contact of the tongue, palatal, and alveolar ridges during the 7th and 8th weeks of gestation (embryological development) and tongue malposition. The second possible theory is remaining amniotic bands, trauma, teratogenic or genetic causes (7,8). In many cases, as in our case, the exact etiology is unknown.

The surgical management of synechia is important, with the main goal being to achieve normal mouth opening so that the patient can reach adequate oral development. This was the case in our patient who underwent surgery as soon as possible.

As mentioned by Mascarella in his paper, most patients with intraoral adhesion bands require tracheostomy, due to an inability to open the mouth and because of upper airway obstruction (2). However, in this case there was no need for tracheostomy.

The extent of the fusion in most synechia cases can be determined by physical examination, and there is no need for further investigation. However, because of difficulties in their airway management, other anomalies should be ruled out.

## Conclusion

Nonisolated congenital intraoral synechia is a rare finding and can present in a wide variety of manifestations, with most cases ultimately needing surgery. Airway management during surgery is very important in such patients. It seems rational to manage patients with face mask-inhalation anesthesia and spontaneous breathing in cases with a low risk of bleeding or where the airway is compromised. Anesthesia and surgery teams should work in cooperation, and emergency tracheostomy devices should be readily available.

## References

[1] Cerrati EW, Ahmed OH, Rickert SM (2015). Isolated congenital maxillomandibular synechiae. Am J Otolaryngol.

[2] Mascarella MA, Schwartz J, Manoukian JJ (2015). Congenital intra-oral adhesions: a surgical approach to cleft palate lateral synechia syndrome. Int J Pediatr Otorhinolaryngol.

[3] Lima LB, Barbosa de Paulo LF, Silva CJ, Mendes VC, Simamoto-Júnior PC, Durighetto AF (2016). Congenital oral synechia and ankyloblepharon filiforme adnatum: Case report and literature review. Int J Pediatr Otorhinolaryngol.

[4] Sybil D, Sagtani A (2013). Cleft palate lateral synechia syndrome. Natl J Maxillofac Surg.

[5] Garca MF, Goktas U, Isik Y, Isik D (2012). Is the coexistence of intraoral synechia and cleft palate anomaly a sequence?. J Craniofac Surg.

[6] Bozdag S, Erdeve O, Konas E, Tuncbilek G, Dilmen U (2011). Management of serious isolated gingival synechia in a newborn: case report and review of the literature. Int J Oral Maxillofac Surg.

[7] German M, Wong H (2011). Oral synechia with epithelial cyst in neonate with cleft palate: a case report. Cleft Palate Craniofac J.

[8] Haydar SG, Tercan A, Uckan S, Gurakan B (2003). Congenital gum synechiae as an isolated anomaly: a case report. J Clin Pediatr Dent.

